# Is Food Insecurity Associated with HIV Risk? Cross-Sectional Evidence from Sexually Active Women in Brazil

**DOI:** 10.1371/journal.pmed.1001203

**Published:** 2012-04-10

**Authors:** Alexander C. Tsai, Kristin J. Hung, Sheri D. Weiser

**Affiliations:** 1 Robert Wood Johnson Health and Society Scholars Program, Harvard University, Cambridge, Massachusetts, United States of America; 2 Center for Global Health, Massachusetts General Hospital, Boston, Massachusetts, United States of America; 3 Department of Obstetrics and Gynecology, Beth Israel Deaconess Medical Center, Boston, Massachusetts, United States of America; 4 Division of HIV/AIDS, San Francisco General Hospital, University of California at San Francisco, San Francisco, California, United States of America; London School of Hygiene & Tropical Medicine, United Kingdom

## Abstract

**Background:**

Understanding how food insecurity among women gives rise to differential patterning in HIV risks is critical for policy and programming in resource-limited settings. This is particularly the case in Brazil, which has undergone successive changes in the gender and socio-geographic composition of its complex epidemic over the past three decades. We used data from a national survey of Brazilian women to estimate the relationship between food insecurity and HIV risk.

**Methods and Findings:**

We used data on 12,684 sexually active women from a national survey conducted in Brazil in 2006–2007. Self-reported outcomes were (a) consistent condom use, defined as using a condom at each occasion of sexual intercourse in the previous 12 mo; (b) recent condom use, less stringently defined as using a condom with the most recent sexual partner; and (c) itchy vaginal discharge in the previous 30 d, possibly indicating presence of a sexually transmitted infection. The primary explanatory variable of interest was food insecurity, measured using the culturally adapted and validated Escala Brasiliera de Segurança Alimentar. In multivariable logistic regression models, severe food insecurity with hunger was associated with a reduced odds of consistent condom use in the past 12 mo (adjusted odds ratio [AOR] = 0.67; 95% CI, 0.48–0.92) and condom use at last sexual intercourse (AOR = 0.75; 95% CI, 0.57–0.98). Self-reported itchy vaginal discharge was associated with all categories of food insecurity (with AORs ranging from 1.46 to 1.94). In absolute terms, the effect sizes were large in magnitude across all outcomes. Underweight and/or lack of control in sexual relations did not appear to mediate the observed associations.

**Conclusions:**

Severe food insecurity with hunger was associated with reduced odds of condom use and increased odds of itchy vaginal discharge, which is potentially indicative of sexually transmitted infection, among sexually active women in Brazil. Interventions targeting food insecurity may have beneficial implications for HIV prevention in resource-limited settings.

*Please see later in the article for the Editors' Summary*

## Abstract 2

Alexander Tsai and colleagues show that in sexually active women in Brazil severe food insecurity with hunger was positively associated with symptoms potentially indicative of sexually transmitted infection and with reduced odds of condom use.

## Editors' Summary

**Background:**

At the beginning of the AIDS epidemic, more men than women were infected with HIV, the virus that causes AIDS, but currently half of all HIV-positive adults are women. Most women become infected with HIV through unprotected sexual intercourse with an infected male partner. Biologically, women are twice as likely to become infected through unprotected heterosexual intercourse as men. Moreover, women are often unable to negotiate condom use because of unequal gender relations—men can insist on unprotected sexual intercourse in many relationships. Another factor often related to unequal gender relations that may shape women's risk of exposure to HIV is food insecurity—limited or uncertain access to enough nutritionally adequate and safe food for an active, healthy life. Recent studies done in sub-Saharan Africa suggest that food insecurity can affect women's engagement in risky sexual behaviors such as unprotected sex, transactional sex (sexual relationships that involve the giving of goods or services such as free lodgings), and commercial sex work.

**Why Was This Study Done?:**

Policymakers planning HIV prevention strategies in resource-limited settings need to know whether food insecurity affects sexual risk taking among women. If it increases risk taking, then interventions that target food insecurity should improve the effectiveness of HIV prevention strategies. However, little is known about food insecurity and sexual risk taking outside sub-Saharan Africa. In this cross-sectional study (a study that characterizes a population at a single point in time), the researchers investigate whether food insecurity is associated with risky sexual behavior among sexually active women in Brazil, a country where the number of new heterosexually transmitted HIV infections among women is increasing. Condom promotion is the mainstay of Brazil's HIV prevention strategy, but less than half of the population reports the use of a condom whenever sexual intercourse occurs (consistent condom use) or at last sexual intercourse (recent condom use), and a greater proportion of men than women report condom use, possibly because of unequal power relations between men and women.

**What Did the Researchers Do and Find?:**

The researchers obtained data on consistent condom use, recent condom use, and self-reported itchy vaginal discharge in the previous 30 days (used here as an indication that a woman may have a sexually transmitted infection) for 12,684 sexually active women from a national survey conducted in Brazil in 2006–2007. They then used multivariable logistic regression (a statistical method) to investigate the association between these outcomes and food insecurity, which was measured using the Escala Brasiliera de Insegurança Alimentar, an 18-item questionnaire that asks people to recall information about the quantity and quality of food available to them over the previous three months. Severe food insecurity with hunger (the most extreme category of food insecurity) was associated with an adjusted odds ratio (AOR) for consistent condom use of 0.67. That is, women who reported severe food insecurity were two-thirds as likely to use a condom whenever they had sexual intercourse as women who were food secure, after adjustment for other factors that might have affected condom use. The probability of consistent condom use was 15% among women who were food secure but only 10.5% among women who had the worst food security. Severe food insecurity with hunger was also associated with a reduced odds of recent condom use (AOR = 0.75), whereas all categories of food insecurity increased the odds of a recent itchy vaginal discharge.

**What Do These Findings Mean?:**

These findings indicate that severe food insecurity with hunger is associated with reduced condom use and with increased occurrence of symptoms that may indicate sexually transmitted disease among sexually active women in Brazil. Because the study looked at women at only a single time point, these findings do not show that food insecurity causes risky sexual behavior. Moreover, these findings may not be generalizable to other settings, and they do not distinguish between regular condom use with a regular partner and regular condom use with casual partners. Also, although the researchers investigated two hypothesized explanations—lack of control in sexual relations and chronic energy deficiency—neither of these factors could explain why food insecurity is associated with risky sexual behavior. Nevertheless, these findings suggest that interventions that target sexual risk reduction behaviors are unlikely to be optimally effective if food insecurity is not taken into account, and, thus, the researchers conclude, HIV prevention strategies in Brazil should include interventions that target food insecurity.

**Additional Information:**

Please access these web sites via the online version of this summary at http://dx.doi.org/10.1371/journal.pmed.1001203.

- Information is available from the US National Institute of Allergy and Infectious Diseases on all aspects of HIV infection and AIDS
- NAM/aidsmap provides basic information about HIV/AIDS, and summaries of recent research findings on HIV care and treatment (in several languages)
- Information is available from Avert, an international AIDS charity on many aspects of HIV/AIDS, including detailed information on HIV and AIDS prevention, women, HIV, and AIDS, and HIV and AIDS in Brazil (in English and Spanish); personal stories of women living with HIV are available
- HIV InSite provides comprehensive and up-to-date information on all aspects of HIV/AIDS from the University of California at San Francisco
- Additional patient stories about living with HIV/AIDS are available through the charity website Healthtalkonline
- A primer on food security from the Food and Agriculture Organization of the United Nations is available
- Information about the 2006–2007 Brazilian national survey on health in women and children is available in Portuguese; a profile of food security in Brazil is also available (some information in English but mainly in Portuguese)

## Introduction

Since the early stages of the HIV epidemic, social science researchers have described how unequal gender relations and gendered structural constraints facilitate the spread of HIV among women [1], particularly among women in sub-Saharan Africa [2]–[5]. A series of newer studies have highlighted food insecurity as a central variable shaping women's risks of HIV exposure. Although women often occupy a primary role in household food production in sub-Saharan Africa, gender bias in the distribution of resources within the household places them at elevated risk for food insecurity compared with men [6],[7]. Qualitative research suggests that inadequate or uncertain access to food exerts an undue influence on women's decisions to engage in transactional sex or unprotected sex [8] or enter commercial sex work [9]. In a population-based study of women in Botswana and Swaziland, food insufficiency was associated with risky sexual behaviors including inconsistent condom use, even after statistical adjustment for education and household income [10],[11], and subsequent studies have replicated these findings in different settings in sub-Saharan Africa [12],[13].

Less is known about power relations, food insecurity, and sexual risk in Brazil, which has undergone successive changes in the gender and socio-geographic composition of its complex epidemic over the past three decades. Although the overall HIV incidence rate stabilized in the 1990s, this trend was driven primarily by reductions in new cases among men [14]. The number of new heterosexually transmitted infections among women has continued to increase, especially among women of reproductive age [14],[15]. Population-based data in Brazil suggest that knowledge about HIV prevention practices is well disseminated, but less than one-half of the population reports consistent condom use or condom use at last sexual intercourse (with a far greater proportion of men reporting condom use compared to women) [16],[17]. These differences are worrisome given that condom promotion has been given primary emphasis in Brazilian HIV prevention programming and policy [18] and that cities where the epidemic is most concentrated are generally characterized by the greatest inequalities between men and women [19]. Although some observers have hypothesized that unequal power relations between men and women in Brazil may explain the observed differences in condom use [16],[20],[21], little empirical work has been done to confirm this hypothesis [22].

At the country level, Brazil has achieved a very low score on the United Nations Development Programme's Gender Inequality Index relative to other countries that are considered to be advanced with regards to human development [23], and textbooks and didactic teaching tend to reinforce gender-based stereotypes [24]. Although increasingly gender-equitable legislation has been adopted in Brazil, such as mandatory joint titling of land to couples, landownership by men still exceeds that of women by a ratio of 8∶1 [25]. In addition, violence against women is highly prevalent, particularly in the north and northeast regions of the country [26]. During times of economic adversity, women and girls living in resource-limited settings may experience worse nutritional and health outcomes than men and boys living in the same households [27]–[29]. These outcomes are relevant to the current context, given that Brazilian men exercise considerable decision-making dominance at the household level [30] and favor their sons in the distribution of resources within the household [31]–[33].

A significant methodological weakness of earlier studies linking food insecurity to HIV risk has been their reliance on non-validated measures of food insecurity and a lack of objective measures of nutritional risk [34], as well as failure to consider the specific mechanisms linking food insecurity to HIV risk reduction behaviors. To address these shortcomings, we analyzed data from a large, geographically diverse sample of women in Brazil to determine whether food insecurity is associated with condom use and/or symptoms of sexually transmitted infection, and to discern the mechanisms underlying these associations.

## Methods

### Ethical Review

The data collection procedures for the 2006 Pesquisa Nacional de Demografia e Saúde da Criança e da Mulher (PNDS) were approved by the ICF Macro Institutional Review Board as well as by the Research Ethics Committee of the Sexually Transmitted Diseases/AIDS Reference and Training Center of the Health Secretariat of São Paulo state. All participants provided oral informed consent. Additional details on staff training, pretesting, and other survey procedures are detailed in the PNDS final report [35]. The specific analysis of PNDS data presented in this paper was reviewed by the Harvard School of Public Health Office of Human Research Administration and deemed exempt from full review because it was based on anonymous public-use data with no identifiable information on participants.

### Data Source

The data for this study were drawn from the PNDS, a national study implemented by the Ministério da Saúde from March 11, 2006, to March 5, 2007, with technical assistance from ICF Macro and the US Agency for International Development. The PNDS employed a probabilistic, complex sampling design, and it was designed to be nationally representative of all women of reproductive age (i.e., 15–49 y). Of 17,411 eligible women selected, 15,575 were successfully interviewed, for a response rate of 89.5%. Data on the primary outcomes of interest were obtained only from women who were sexually active. Therefore, the analyses reported in this paper were restricted to women who reported sexual activity with a man in the previous 12 mo.

### Conceptual Framework and Statistical Analysis

**Figure 1:**
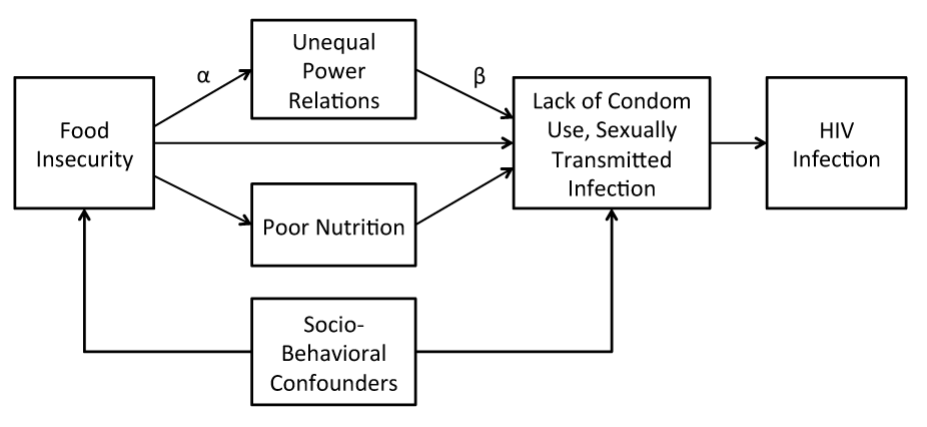
Conceptual framework linking food insecurity to HIV risk. The pathways between food insecurity and HIV risk may be direct or indirect. The indirect effects are mediated through unequal power relations and/or poor nutrition. For each hypothesized mediator, the indirect effect of the exposure on the outcomes (lack of condom use and sexually transmitted infection) is computed as the product of α×β. Other socio-behavioral variables may confound the observed association between food insecurity and HIV risk.

The conceptual framework guiding our analysis, adapted from previously published work [36], is depicted in Figure 1. Our primary focus was to explain HIV risk. Accordingly, the primary outcomes of interest in our analysis were (a) consistent condom use, defined as using a condom at each occasion of sexual intercourse in the previous 12 mo; (b) recent condom use, less stringently defined as using a condom with the most recent sexual partner; and (c) self-report of an itchy vaginal discharge in the previous 30 d, possibly indicating presence of a sexually transmitted infection. The primary explanatory variable of interest was household food insecurity, defined as “access by all people at all times to enough food for an active, healthy life” ([37], p. 1560). To measure food insecurity, we used the Escala Brasiliera de Segurança Alimentar (EBIA) [38],[39], a culturally adapted, Portuguese version of the US Household Food Security Survey Module [40]. Both the US and Brazilian scales differentiate between households with and without children in assessing the degree of food insecurity. The 18-item EBIA scale employs a recall period of 3 mo (compared to 12 mo for the US version) and has demonstrated good internal consistency as well as content, convergent, and internal validity [38],[39]. Using the previously validated algorithm [38],[39], we assigned participants into one of four categories: food secure, food insecure without hunger, moderately food insecure with hunger, and severely food insecure with hunger.

In our conceptual framework, food insecurity may have a direct effect on women's HIV risk, or the effect may be mediated through intervening variables that can also serve as programming or policy levers. In this analysis, we considered two specific mediators, poor nutrition and/or unequal power relations. Food insecurity may undermine women's ability to negotiate for condom use through its effects on nutritional risk and chronic energy deficiency. Previous research has also linked food insecurity to lack of control in sexual relations and to forced sex [10],[11]. In order to investigate these hypotheses, we operationalized chronic energy deficiency as a binary variable equal to one if the participant was underweight (defined as a body mass index <18.5 kg/m^2^ [41]), zero otherwise. As a proxy for unequal power relations, we constructed a variable based on the participant's responses to questions about whether she felt it would be acceptable for a woman to refuse sexual intercourse with her husband or partner in five hypothetical scenarios [42]: if she knew he had a sexually transmitted disease, if she knew that he was having sexual intercourse with other women, if she had given birth to a child recently, if she was tired, or if she did not want to have sexual intercourse. Women who responded “no” to all five scenarios were (stringently) categorized as lacking control in sexual relations.

All analyses were conducted using the Stata statistical software package (version 12.0, StataCorp). To estimate the association between food insecurity and the outcomes of interest, we fit multivariable logistic regression models to the data, with cluster-correlated robust estimates of variance [43]–[45]. This modeling approach appropriately recognizes that variables measured at the level of the primary sampling unit have a smaller effective sample size and corrects the standard errors for potentially correlated observations between participants who live in the same primary sampling unit. We did not use the sampling weights provided by ICF Macro because this analysis was restricted to sexually active women only, and sampling weights were not provided for analyses restricted to this sub-sample of the population.

As depicted in the conceptual framework, socio-behavioral variables may confound the association between food insecurity and sexual risk. In our regression analyses, we therefore adjusted for potential confounding by the following socio-behavioral variables: age, racial/ethnic group (white [*branca*], black [*preta*], mixed [*parda*], Asian [*amarela*], or indigenous [*indigena*]), urban residence, macro-region of the country (north, northeast, southeast, south, or center-west), domestic partnership status (legally or formally married, not married but living with a partner in a consensual union, never married, separated, divorced, or widowed), Catholic religion, news reading frequency (reads the news daily, nearly every day, once per week, less than once per month, does not read), within-country quintiles of household asset wealth [46], and current use of cigarettes.

If simple linear regression models had been used in this analysis, we could have estimated the association between sexual risk and the hypothesized mediator adjusted for food insecurity (depicted as β in the conceptual framework), and then estimated the association between the food insecurity and the hypothesized mediator (α). The indirect effect of food insecurity on sexual risk, i.e., the portion of the effect of food insecurity on sexual risk that is due to the mediating variables of interest, could be computed as the product α×β [47], and the asymptotic variance would be computed using the multivariate delta method [48]. In the context of logistic regression, however, parameter rescaling tends to increase the apparent magnitude of the estimated regression coefficient and counters the effect of including the (potential) socio-behavioral confounders, as noted above. We therefore implemented a previously published algorithm [49],[50] to rescale the parameter estimates in order to decompose the total effect of food insecurity into its indirect and direct effects.

We also investigated whether the effect of food insecurity on the outcomes of interest varied according to domestic partnership status or fertility preferences. Fertility preferences were measured with a binary variable equal to one if the woman expressed a preference for no further childbearing and zero if the woman was undecided or expressed a preference to have more children. To assess potential effect modification, we included both the main effect terms and the interaction terms (with food insecurity) in the regression models and then used Wald-type *F*-tests to determine whether these variables modified the associations between food insecurity and the outcomes of interest. These interaction tests were based on our hypothesis that the adverse effects of food insecurity on condom use may be strongest among women who have a stronger preference for condom use, i.e., women who do not desire to bear more children or women who are not currently in a domestic partnership.

## Results

Of the 15,575 women interviewed for the study, 12,684 (81.4%) reported sexual activity with a man in the previous 12 mo and were therefore included in this analysis. The distributions of food insecurity scores were similar for women who were sexually active and women who were not: the mean EBIA scores were similar (*t* = 0.18; *p* = 0.86), and the percentages of women assigned to the different categories of food insecurity were also similar (*χ^2^* = 1.02; *p* = 0.80). Consistent condom use was reported by 2,210 women (18.0%), condom use at last sexual intercourse was reported by 3,172 women (25.7%), and itchy vaginal discharge was reported by 1,337 women (10.8%). Summary statistics are presented in Table 1. On the EBIA, most women were categorized as food secure (9,343 [73.7%]), while 1,762 women (13.9%) were categorized as food insecure without hunger, 783 (6.2%) were categorized as moderately food insecure with hunger, and 473 (3.7%) were categorized as severely food insecure with hunger. The Cronbach's α for the EBIA was 0.91, indicating a high degree of internal consistency.

**Table 1:** Summary statistics.

Variable	All Participants (*n* = 12,684)	Food Security Category		χ^2^ Test Statistic^a^
		Food Secure, Food Insecure without Hunger, or Moderately Food Insecure with Hunger (*n* = 12,211)	Severely Food Insecure with Hunger (*n* = 473)	
**Age**	32 (24–40)	32 (24–39)	34 (27–41)	20.6***
**Race**				90.4***
White (*branca*)	4,824 (39.4%)	4,725 (40.1%)	99 (21.2%)	
Black (*preta*)	1,210 (9.9%)	1,150 (9.8%)	60 (12.9%)	
Mixed (*parda*)	5,607 (45.8%)	55,334 (45.3%)	273 (58.6%)	
Asian (*amarela*)	342 (2.8%)	334 (2.8%)	8 (1.7%)	
Indigenous (*indigena*)	265 (2.2%)	239 (2.0%)	26 (5.6%)	
**Urban residence**	8,713 (70.5%)	8,432 (70.9%)	281 (59.4%)	29.0***
**Macro-region**				241.8***
North	2,126 (17.2%)	1,940 (16.3%)	186 (39.3%)	
Northeast	2,352 (19.0%)	2,218 (18.7%)	134 (28.3%)	
Southeast	2,627 (21.3%)	2,567 (21.6%)	160 (12.7%)	
South	2,713 (22.0%)	2,675 (22.5%)	38 (8.0%)	
Center-west	2,543 (20.6%)	2,488 (20.9%)	55 (11.6%)	
**Domestic partnership status**				68.1***
Other	2,779 (22.5%)	2,673 (22.5%)	106 (22.4%)	
Cohabiting	4,181 (33.8%)	3,944 (33.2%)	237 (50.1%)	
Married	5,401 (43.7%)	5,271 (44.3%)	130 (27.5%)	
**Catholic religion**	10,248 (82.9%)	9,853 (82.9%)	395 (83.5%)	0.13
**Frequency of reading the news**				167.5***
Does not read	4,468 (36.2%)	4,174 (35.1%)	294 (62.2%)	
Less than once a month	2,363 (19.1%)	2,274 (19.1%)	89 (18.8%)	
At least once a week	2,773 (22.5%)	2,715 (22.9%)	58 (12.3%)	
Nearly every day	1,546 (12.5%)	1,527 (12.9%)	19 (4.0%)	
Daily	1,201 (9.7%)	1,188 (10.0%)	13 (2.8%)	
**Household asset wealth index**				550.5***
Most poor	2,501 (20.3%)	2,200 (18.7%)	281 (59.8%)	
Very poor	2,514 (20.4%)	2,398 (20.2%)	116 (24.7%)	
Poor	2,372 (19.2%)	2,319 (19.6%)	53 (11.3%)	
Less poor	2,517 (20.4%)	2,498 (21.1%)	19 (4.0%)	
Least poor	2,426 (19.7%)	2,425 (20.5%)	1 (0.2%)	
**Smokes cigarettes**	1,881 (15.2%)	1,768 (14.9%)	113 (23.9%)	28.7***

All data are number (percent), except for age, which is median (interquartile range).

a Represents the result of a non-parametric K-sample test on the equality of medians with continuity correction (for continuous variables) or Pearson's chi-squared test (for categorical variables).

***: Statistical significance at the level of *p*<0.001.

In multivariable analyses, severe food insecurity with hunger was associated with a statistically significant reduced odds of consistent condom use (adjusted odds ratio [AOR] = 0.67; 95% CI, 0.48–0.92) and condom use at last sexual intercourse (AOR = 0.84; 95% CI, 0.81–0.86) (Table 2). The estimated odds ratios for food insecurity categories of lesser severity did not have statistically significant associations with the condom use outcomes, whereas all categories of food insecurity were associated with increased odds of reporting symptoms of itchy vaginal discharge. These estimated associations were also large in magnitude: evaluated at the mean of the other covariates, changing food security status from food secure to severely food insecure with hunger resulted in a change of the predicted probability of consistent condom use from 15.0% to 10.5%, while the predicted probability of self-reported itchy vaginal discharge changed from 9.2% to 16.4%. A number of other important patterns were also evident. There was little evidence of racial/ethnic differences. Condom use and self-reported itchy vaginal discharge were more likely among women in the north and northeast regions of the country, which have been less affected by the HIV epidemic and have experienced a slower rise in HIV incidence [14]. Consistent with previous work [51], greater reading frequency was associated with greater odds of condom use and reduced odds of self-reported itchy vaginal discharge.

**Table 2:** Associations between food insecurity and sexual risk outcomes.

Variable	Consistent Condom Use in the Past 12 mo		Condom Use at Last Sexual Intercourse		Itchy Vaginal Discharge in the Past 30 d	
	OR (95% CI)	AOR (95% CI)	OR (95% CI)	AOR (95% CI)	OR (95% CI)	AOR (95% CI)
**Food insecurity category**						
Food secure	Ref	Ref	Ref	Ref	Ref	Ref
Food insecure without hunger	**0.85 (0.74–0.99)**	0.94 (0.79–1.10)	0.93 (0.82–1.04)	1.00 (0.87–1.14)	**1.61 (1.37–1.89)**	**1.46 (1.24–1.73)**
Moderately food insecure with hunger	0.88 (0.73–1.07)	1.00 (0.81–1.24)	0.86 (0.73–1.02)	0.95 (0.78–1.15)	**1.94 (1.58–2.37)**	**1.78 (1.44–2.22)**
Severely food insecure with hunger	**0.54 (0.40–0.72)**	**0.67 (0.48–0.92)**	**0.60 (0.47–0.77)**	**0.75 (0.57–0.98)**	**2.16 (1.67–2.79)**	**1.94 (1.47–2.56)**
**Age (in 5-y blocks)**	**0.77 (0.75–0.79)**	**0.87 (0.90–0.96)**	**0.75 (0.73–0.77)**	**0.84 (0.81–0.86)**	**0.95 (0.93–0.98)**	**0.95 (0.92–0.98)**
**Race**						
White	1.09 (0.98–1.22)	1.10 (0.97–1.24)	0.97 (0.88–1.07)	0.96 (0.86–1.08)	**0.81 (0.72–0.92)**	0.95 (0.83–1.10)
Black	1.09 (0.92–1.28)	1.06 (0.88–1.26)	1.03 (0.89–1.19)	0.97 (0.83–1.14)	0.96 (0.79–1.18)	0.98 (0.80–1.21)
Mixed	Ref	Ref	Ref	Ref	Ref	Ref
Asian	1.24 (0.95–1.63)	0.96 (0.71–1.30)	1.19 (0.93–1.53)	0.92 (0.68–1.24)	0.82 (0.56–1.21)	0.88 (0.58–1.33)
Indigenous	1.22 (0.87–1.70)	1.14 (0.83–1.57)	1.15 (0.84–1.57)	1.09 (0.80–1.49)	0.82 (0.53–1.27)	0.73 (0.45–1.18)
**Urban residence**	**1.75 (1.54–1.99)**	1.14 (1.00–1.30)	**1.89 (1.69–2.12)**	**1.31 (1.15–1.49)**	**0.78 (0.69–0.88)**	0.94 (0.82–1.08)
**Macro-region**						
North	Ref	Ref	Ref	Ref	Ref	Ref
Northeast	**0.78 (0.65–0.94)**	0.85 (0.71–1.02)	0.94 (0.80–1.12)	1.06 (0.89–1.25)	**0.68 (0.56–0.83)**	**0.69 (0.56–0.84)**
Southeast	0.87 (0.73–1.03)	**0.77 (0.64–0.92)**	0.86 (0.73–1.02)	0.85 (0.71–1.01)	**0.70 (0.58–0.85)**	0.87 (0.72–1.07)
South	0.84 (0.70–1.00)	**0.75 (0.62–0.91)**	0.87 (0.73–1.02)	0.87 (0.73–1.05)	**0.71 (0.59–0.86)**	0.91 (0.74–1.12)
Center-west	**0.81 (0.68–0.97)**	**0.76 (0.63–0.91)**	0.86 (0.73–1.02)	0.86 (0.72–1.02)	**0.77 (0.65–0.93)**	0.90 (0.75–1.08)
**Domestic partnership status**						
Other	Ref	Ref	Ref	Ref	Ref	Ref
Cohabiting	**0.16 (0.15–0.19)**	**0.20 (0.18–0.23)**	**0.18 (0.16–0.20)**	**0.22 (0.19–0.25)**	**1.39 (1.20–1.63)**	**1.23 (1.05–1.44)**
Married	**0.15 (0.13–0.17)**	**0.19 (0.17–0.22)**	**0.14 (0.12–0.16)**	**0.19 (0.17–0.22)**	1.12 (0.96–1.31)	1.16 (0.98–1.37)
**Catholic religion**	**0.87 (0.77–0.97)**	1.00 (0.88–1.14)	**0.88 (0.79–0.98)**	1.04 (0.92–1.17)	0.93 (0.80–1.08)	0.94 (0.81–1.10)
**Frequency of reading the news**						
Does not read	Ref	Ref	Ref	Ref	Ref	Ref
Less than once a month	**1.60 (1.40–1.84)**	**1.34 (1.16–1.55)**	**1.54 (1.37–1.74)**	**1.29 (1.14–1.47)**	0.92 (0.79–1.07)	0.91 (0.77–1.06)
At least once a week	**1.91 (1.67–2.17)**	**1.38 (1.20–1.60)**	**1.86 (1.66–2.09)**	**1.39 (1.22–1.58)**	**0.71 (0.60–0.82)**	**0.78 (0.66–0.91)**
Nearly every day	**2.47 (2.13–2.86)**	**1.61 (1.36–1.91)**	**2.41 (2.11–2.75)**	**1.64 (1.41–1.91)**	**0.59 (0.49–0.73)**	**0.74 (0.60–0.91)**
Daily	**2.57 (2.19–3.01)**	**1.61 (1.33–1.94)**	**2.51 (2.17–2.90)**	**1.67 (1.41–1.98)**	**0.51 (0.40–0.64)**	**0.64 (0.50–0.84)**
**Household asset wealth index**						
Most poor	Ref	Ref	Ref	Ref	Ref	Ref
Very poor	**1.46 (1.23–1.72)**	**1.39 (1.15–1.68)**	**1.44 (1.26–1.66)**	**1.41 (1.20–1.66)**	0.88 (0.75–1.05)	1.01 (0.84–1.22)
Poor	**1.46 (1.26–1.71)**	**1.36 (1.13–1.65)**	**1.39 (1.21–1.60)**	**1.33 (1.12–1.57)**	**0.79 (0.66–0.94)**	1.02 (0.83–1.25)
Less poor	**1.61 (1.36–1.90)**	**1.46 (1.20–1.78)**	**1.46 (1.26–1.68)**	**1.39 (1.18–1.65)**	**0.80 (0.67–0.95)**	1.11 (0.90–1.37)
Least poor	**2.05 (1.74–2.42)**	**1.50 (1.21–1.86)**	**1.83 (1.59–2.11)**	**1.43 (1.18–1.73)**	**0.45 (0.37–0.55)**	**0.73 (0.56–0.94)**
**Smokes cigarettes**	0.96 (0.85–1.09)	0.93 (0.81–1.07)	**0.88 (0.79–0.99)**	**0.84 (0.74–0.96)**	0.90 (0.77–1.05)	**0.84 (0.71–0.98)**

Bold indicates statistical significance at the level of *p*<0.05.

OR, odds ratio; Ref, reference.

In the mediation analyses, neither underweight nor lack of control in sexual relations proved to be substantive mediators of the relationship between food insecurity and the outcomes of interest (Table 3). Across the outcomes of interest, when these hypothesized mediators were included in the multivariable regression models, the estimated AOR for severe food insecurity changed minimally and even shifted away from the null. In addition, we assessed effect modification by domestic partnership status and fertility preferences. No statistically significant effect modification was observed for any of the outcomes, although severe food insecurity appeared to have the strongest association with condom use and symptoms of sexually transmitted infection among women who prefer more children.

**Table 3:** Mediation and effect modification analyses.

Variable	Consistent Condom Use in the Past 12 mo	Condom Use at Last Sexual Intercourse	Itchy Vaginal Discharge in the Past 30 d
**Mediation analysis**			
Unadjusted effect of severe food insecurity with hunger	**OR = 0.55 (95% CI, 0.41–0.74)**	**OR = 0.62 (95% CI, 0.48–0.79)**	**OR = 1.89 (95% CI, 1.47–2.43)**
Adjusted effect^a^	**AOR = 0.68 (95%CI, 0.49–0.94)**	**AOR = 0.76 (95% CI, 0.57–0.99)**	**AOR = 1.52 (95% CI, 1.16–2.01)**
Adjusted effect, accounting for underweight^a^	**AOR = 0.68 (95% CI, 0.49–0.94)**	**AOR = 0.75 (95% CI, 0.57–0.99)**	**AOR = 1.53 (95% CI, 1.17–2.01)**
Percentage of total effect due to underweight	−0.9%	−2.7%	−0.8%
Adjusted effect, accounting for lack of control in sexual relations^a^	**AOR = 0.67 (95% CI, 0.49–0.93)**	**AOR = 0.76 (95% CI, 0.58–0.99)**	**AOR = 1.59 (95% CI, 1.21–2.08)**
Percentage of total effect due to lack of control in sexual relations	−2.9%	−2.4%	−1.5%
**Effect modification analysis**			
**By domestic partnership status**			
Among non-partnered women	AOR = 0.68 (95% CI, 0.43–1.07)	AOR = 0.71 (95% CI, 0.47–1.08)	**AOR = 2.46 (95% CI, 1.41–4.30)**
Among cohabiting women	AOR = 0.69 (95% CI, 0.39–1.21)	AOR = 0.75 (95% CI, 0.50–1.13)	**AOR = 1.60 (95% CI, 1.07–2.39)**
Among married women	AOR = 0.44 (95% CI, 0.16–1.23)	AOR = 0.79 (95% CI, 0.41–1.53)	**AOR = 2.22 (95% CI, 1.34–3.67)**
Wald-type *F*-test (*p*-value)	5.49 (0.48)	3.46 (0.75)	6.60 (0.36)
**By fertility preference**			
Among women who prefer no more children	AOR = 0.73 (95% CI, 0.46–1.16)	AOR = 0.76 (95% CI, 0.52–1.10)	**AOR = 1.81 (95% CI, 1.14–2.88)**
Among women who prefer more children/undecided	**AOR = 0.54 (95% CI, 0.34–0.87)**	**AOR = 0.65 (95% CI, 0.43–0.98)**	**AOR = 2.03 (95% CI, 1.46–2.82)**
Wald-type *F*-test (*p*-value)	0.20 (0.90)	0.67 (0.72)	0.36 (0.83)

a Regression estimates adjusted for age, race, urban residence, macro-region, domestic partnership status, Catholic religion, news reading frequency, household asset wealth, and cigarette use. Bold indicates statistical significance at the level of *p*<0.05.

## Discussion

Using data on 12,684 sexually active women sampled from diverse geographic regions of Brazil, we found that condom use was infrequent and that severe food insecurity with hunger was associated with reduced odds of condom use and increased odds of self-reported itchy vaginal discharge, possibly indicating presence of a sexually transmitted infection. These estimated associations were statistically significant, large in magnitude, and robust to statistical adjustment for known confounders. Given the infrequency of condom use among Brazilian women, a finding echoed by previous studies [16]–[18], and the centrality of condom promotion to Brazil's HIV prevention strategy, our findings have important implications for policy and programming for HIV prevention.

It is well known that the social and economic marginalization of women constrains their ability to engage in HIV risk reduction behaviors [1]–[5]. Newer research has specifically identified food insecurity as a critical variable influencing women's risks of sexual violence [11] and exposure to HIV [10],[12],[13]. In these studies, however, food insecurity was measured using just one [10]–[12] or two [13] questions about food insufficiency. Food insecurity is a complex, multidimensional phenomenon characterized not only by insufficient food intake, but also by poor diet quality, disrupted eating patterns, and anxiety and uncertainty about access [52]. The single-question item incorporated into the US National Center for Health Statistics' Third National Health and Nutrition Examination Survey has demonstrated poor sensitivity for identifying food insecure households [53]. What is new about our analysis is our use of a well-developed, culturally adapted 18-item food insecurity scale that measures the entire range of human experience with food insecurity, from food security to severe food insecurity with hunger [38],[39].

We investigated two hypothesized mediators, underweight and lack of control in sexual relations, but these variables did not yield a substantive degree of mediation. This suggests that the observed association is due to a direct effect of food insecurity on sexual risk, or that it is due to mediation by unmeasured variables such as depression and other negative affect states [54] or condom use self-efficacy [55],[56]. In addition, measurement error in the variable for lack of control in sexual relations may have undermined our ability to adequately test for evidence of mediation. We did not have access to better developed measurements, such as the sexual relationship power scale [57]. However, even if the precise mechanism of action remains unknown, if the observed association is causal then food security interventions could still have beneficial effects on women's sexual risk, irrespective of the mechanism of action.

Our findings about food insecurity and sexual risk add to the burgeoning research base that highlights the importance of food insecurity as a variable of central importance in HIV prevention efforts [58],[59]. In Brazil, the specific targeting of high-risk women through food supplementation or livelihood interventions may help to equalize gender-based intra-household bargaining power differentials. Specifically, microfinance-based interventions have been promoted for reducing HIV risk [60],[61]. However, in Brazil, women have not been the traditional focus of microfinance initiatives as in other countries like India and Bangladesh [62].

Several limitations must be considered in interpreting our findings. First, we did not use the sampling weights provided by ICF Macro. Our analysis was restricted to sexually active women, a sub-sample for which sampling weights were not provided, and application of the weights for national representativeness could lead to unpredictable biases. This would not necessarily be considered as a source of potential bias, however, given that we do not attempt to generalize our findings to the population of Brazilian women of reproductive age. The large sample size does make our analysis to our knowledge the largest study of its kind to date, suggesting broad applicability across diverse socio-demographic groups in an emerging economy.

Second, measurement error in the outcomes of interest could bias our estimates in unpredictable ways. Although condom use at last sexual intercourse may be erroneously measured [63], consistent condom use signifies a greater degree of commitment and intention and is less subject to errors in reporting [64]. In addition, itchy vaginal discharge may be symptomatic of other conditions that are not sexually transmitted (e.g., genital/vulvovaginal candidiasis). In a meta-analysis of symptoms and signs of chlamydial infection and gonorrhea among women, the specificity of vaginal itching and vaginal discharge ranged from 65% to 79%, depending on the study setting [65]. Random measurement error in the dependent variable would have biased our estimates towards the null and resulted in more conservative estimates of association, however. In order for systematic measurement error in the dependent variable to bias our estimates away from the null, the systematic measurement error would need to be somehow related to the exposure of interest (food insecurity). Studies of food insecurity and HIV risk clearly warrant the collection of biomarker data, but these data are more difficult to obtain compared to measures of self-report. Because of the stigma attached to HIV, household surveys that incorporate HIV testing have typically experienced a 10%–20% lower response rate to HIV testing than to the household survey modules [66].

Third, study participants' mental health was not assessed. A cross-sectional study of women living in Goa, India, demonstrated that complaints of vaginal discharge were associated with both hunger and non-psychotic psychiatric morbidity [67],[68]. The study authors interpreted the latter finding as indicative of vaginal discharge as a bodily idiom of distress. Non-psychotic psychiatric morbidity could be potentially considered an unmeasured confounder with regards to our analysis. However, in light of prior studies linking food insecurity to depression and other markers of psychological distress [69]–[72], we believe that including such a variable in our regression models (if it had been available) would have resulted in over-adjustment by conditioning on part of the effect of interest.

Fourth, the observed associations between food insecurity and the outcomes could also be explained by unmeasured confounding. Our multivariable models included statistical adjustment for key variables known to confound the relationship between food insecurity and sexual risk (e.g., educational attainment and economic status) but may have omitted others. Most notably, we were unable to distinguish between consistent condom use with regular partners versus consistent condom use with casual partners [73]. There may have been differential patterning of food insecurity and condom use by partner type [74], given the highly negative meanings that may be attached to condom use in the context of marital relationships or regular sexual partnerships [1],[75]. Because casual or transactional partnerships are more likely to be economically motivated and characterized by greater frequency of HIV transmission risk behaviors [76],[77], failure to account for the type of partner could have confounded our estimates of the association between food insecurity and sexual risk. However, in our data we found no evidence of effect modification by domestic partnership status, and we would expect domestic partnership to be correlated (however weakly) with a lower propensity to have casual sexual partners.

Fifth, both the exposure and outcomes of interest were based on participant self-report. If study participants who provided responses consistent with more severe levels of food insecurity were also more likely to under-report condom use or over-report itchy vaginal discharge, this could have biased our estimates away from the null.

Sixth, the direction of causality is generically uncertain with data of a cross-sectional nature. However, the estimated associations presented in our analysis are strong, increasing with the intensity of the exposure, consistent with previously published research conducted in independent samples [10],[12],[13], plausible, and coherent with our socio-cultural understanding of the Brazilian context. Together these elements suggest our conservative interpretation of the data is correct [78], but longitudinal or experimental study designs in future work would help to strengthen claims of causality.

In summary, this study presents evidence from sexually active women living in Brazil that food insecurity is associated with reduced use of condoms during sexual intercourse. If the estimated association is causal, our findings suggest that interventions targeting food insecurity may have beneficial implications for HIV prevention. Individual-level cognitive and/or behavioral interventions targeting HIV risk avoidance or risk reduction behaviors are likely to be less than optimally effective if these structural factors are not also taken into account.

## Abbreviations

AOR: adjusted odds ratio
EBIA: Escala Brasiliera de Segurança Alimentar
PNDS: Pesquisa Nacional de Demografia e Saúde da Criança e da Mulher
